# JOURNAL OF GERONTOLOGY SOCIAL SCIENCES: FEATURED 2023 EDITOR’S CHOICE ARTICLES

**DOI:** 10.1093/geroni/igae098.1188

**Published:** 2024-12-31

**Authors:** Jessica Kelley

**Affiliations:** Chair

## Abstract

Social science inquiry on age, aging, and the life course spans many topics and methodologies. This symposium highlights papers that were selected as Editor’s Choice articles in the Journal of Gerontology Social Sciences in 2023. These papers highlight methodological innovations, important advancements in our state of knowledge in an area, or emerging issues in the study of aging and older adults. Wright et al. compare differences in income pooling between remarried and cohabiting older adults. Taylor et al. present a qualitative study of the ways that kinless older adults navigate a dementia onset. Stokes et al. utilize novel dyadic analysis to explore the interrelationship of loneliness and cognition among married couples over time. Latham Minus et al. document mental and physical health among older adults who are formerly incarcerated, showing differences by gender and race. Al-Rousan et al. examine the relationship between migration history and cognitive functioning among older Arab Americans and refugees.

